# Supplementary material for: Amphiphilic Bowl-Shaped Janus Particles Prepared via Thiol–Ene Click Reaction for Effective Oil–Water Separation
Source: Nanomaterials (Basel). 2023 Jan 22;13(3):455. doi: 10.3390/nano13030455 (PMC9921205; doi:10.3390/nano13030455)
Supplement: Supplementary file 1 [file nanomaterials-13-00455-s001.zip › nanomaterials-2150447-supplementary.pdf]

Supporting Information

Amphiphilic bowl-shaped Janus particles prepared via thiol-ene click reaction for effective oil–water separation

Xian Qi, Yaxian Du, Ziqiang Zhang and Xu Zhang *

National-Local Joint Engineering Laboratory for Energy Conservation of Chemical Process Integration and Resources Utilization, School of Chemical Engineering and Technology, Hebei University of Technology, Tianjin 300401, China

* Correspondence: xuzhang@hebut.edu.cn

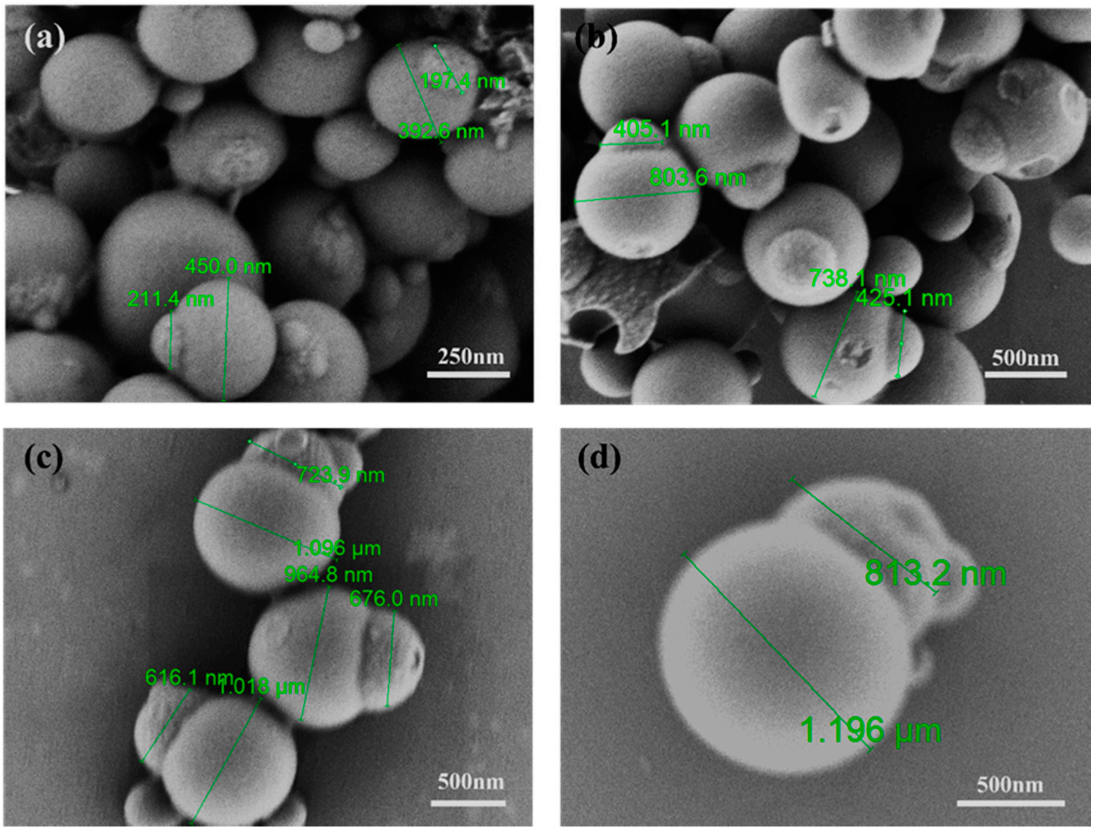

Figure S1. The SEM images of SiO_2@MPSQ particles prepared from silica templates with different particle sizes:(a) 243 nm,(b) 426 nm,(c) 550 nm and (d) 760 nm

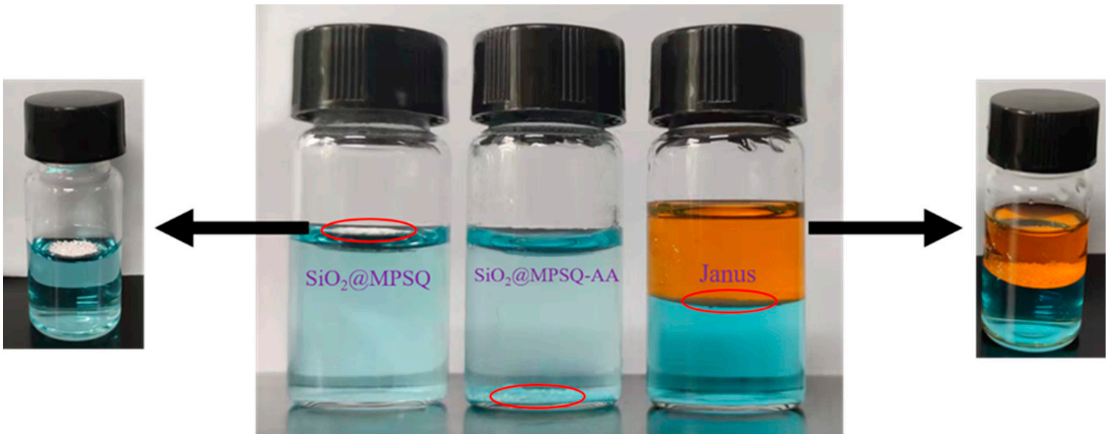

Figure S2. Diagram of three particles in liquid (yellow for oil phase, blue for water phase)

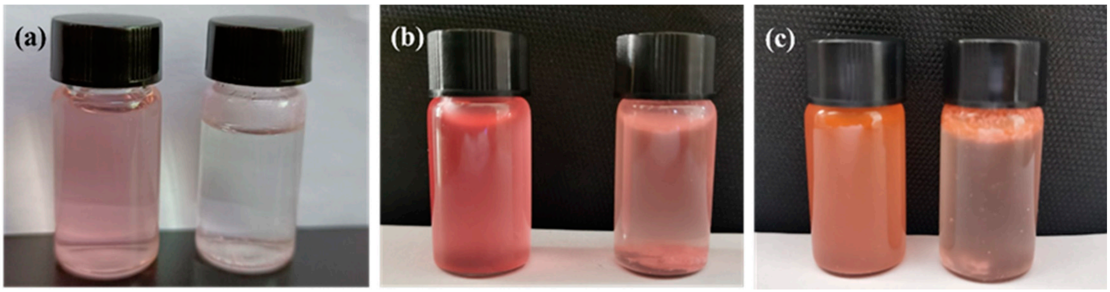

Figure S3. Photograph of separation of n-heptane emulsions with different concentrations mediated by Janus particles: (a) 0.5%, (b) 5%, (c) 7%

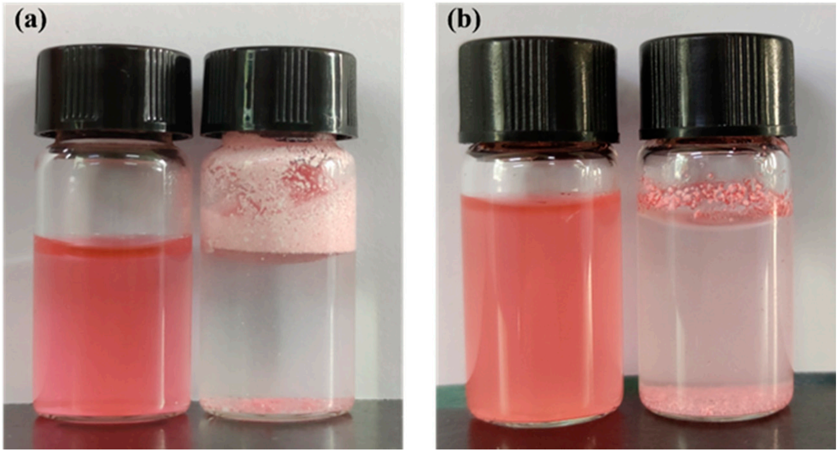

Figure S4. Photograph characterization of Janus particle-mediated separation of toluene (a) and cyclohexane (b) emulsions at the same concentration (3%)
